# Suppression of certain intestinal microbiota metabolites may lead to gestational diabetes in mice fed a high-fat diet

**DOI:** 10.3389/fmicb.2024.1473441

**Published:** 2024-09-16

**Authors:** Ya-ping Xie, Hui-fen Zhao, Shu Lin, Xian-long Wang, Yi-fei Liu, Bao-yuan Xie

**Affiliations:** ^1^Nursing Department, The Second Affiliated Hospital of Fujian Medical University, Quanzhou, China; ^2^Centre of Neurological and Metabolic Research, The Second Affiliated Hospital of Fujian Medical University, Quanzhou, China; ^3^Group of Neuroendocrinology, Garvan Institute of Medical Research, Sydney, NSW, Australia; ^4^Department of Bioinformatics, Fujian Key Laboratory of Medical Bioinformatics, School of Medical Technology and Engineering, Fujian Medical University, Fuzhou, China; ^5^Central Laboratory, The Second Affiliated Hospital of Fujian Medical University, Quanzhou, China

**Keywords:** gestational diabetes mellitus, intestinal microbiota, exosomes, high-fat diet, short-chain fatty acids

## Abstract

**Background:**

We aim to establish a gestational diabetes mellitus (GDM) mouse model with mice fed with a high-fat diet (HFD) in comparison with pregnant mice with normal blood glucose levels to investigate the role of intestinal microbiota in the development of HFD-induced GDM.

**Methods:**

We divided healthy 6-week-old female C57BL mice into an HFD-induced GDM group and a normal diet group. Their bacterial flora and metabolites in intestinal fecal exosomes were co-analyzed using 16 s multi-region sequencing and compared.

**Findings:**

Alpha (α) diversity was lower within the model group compared to the control group. Beta (β) diversity was significantly different between the two groups. The relative abundances of *Lactobacillus*, *Actinomyces*, *Rothia*, and Bacteroidetes were significantly different between the two groups. Fermentation and nitrate consumption were significantly higher in the GDM group. Multiple bacteria were associated with glycerophosphocholine, S-methyl-5′-thioadenosine, quinolinate, galactinol, deoxyadenosine, DL-arginine, and 2-oxoadenic acid.

**Interpretation:**

Imbalances in the production of *Lactobacillus*, Bacteroidetes, *Actinomyces*, and *Rothia* and their related metabolites may lead to metabolic disturbances in GDM. These indicators may be used to assess changes affecting the intestinal microbiota during pregnancy and thus help modulate diet and alter blood glucose.

## 1 Introduction

Gestational diabetes mellitus (GDM) is an abnormality of glucose tolerance detected for the first time in pregnant women. Among women of childbearing age (20–49 years), 16.2% had some type of hyperglycemia during pregnancy, and GDM is approximately 90–95% of those cases (Sklempe Kokic et al., 2018; Gestational Diabetes Mellitus, 2017). Currently, the yearly increase in the incidence of GDM places a serious burden on public healthcare organizations (Zhu and Zhang, 2016). GDM may interfere with the normal course of pregnancy and labor. It increases the incidence of adverse pregnancy outcomes, such as pre-eclampsia and preterm labor (Draznin et al., 2022). Their offspring are two to eight times more likely to develop kidney disease and diabetes mellitus than healthy pregnant women (Damm et al., 2016). Current evidence suggests that the mechanisms of GDM include placental dysfunction, β-cell dysfunction, inflammation, and insulin resistance (Li et al., 2019; Tanaka et al., 2021), and are also influenced by environmental factors. A sedentary lifestyle and a high-fat diet (HFD) may lead to the development of GDM (Jiang et al., 2019; Yong et al., 2020). However, the mechanism by which HFD increases the risk of GDM remains unclear.

Fecal extracellular vesicles isolated from mice fed highly saturated fatty acids slowed glucose metabolism through insulin resistance in skeletal muscle and adipose tissue (Choi et al., 2015). However, their specific mechanisms of action in extracellular vesicles are unknown. Exosomes, a type of extracellular vesicle derived from nuclear endosomes, are important mediators of intercellular communication. They transport a wide range of biologically active molecules, such as non-coding RNAs, messenger RNAs, and proteins (Woith et al., 2019), which regulate inflammation, metabolism, and intestinal barrier function (Kamerkar et al., 2017; Colombo et al., 2014; Díez-Sainz et al., 2022). Additionally, a previous study focusing on the differences in the intestinal microbiota between healthy pregnant women and those with GDM found that *Alistipes putredinis*, *Coprococcus*, Firmicutes, and Bacteroidetes may be associated with the development of GDM (Sun et al., 2023; Vavreckova et al., 2022; Yan et al., 2022; Liu et al., 2023). Thus, exosomes may be the key mediators of intestinal microbiota and host communication. An HFD may induce dysbiosis of the intestinal microbiota through alterations in the morphology of extracellular vesicles within the intestinal microbiota, which can have a deleterious effect on energy homeostasis (Díez-Sainz et al., 2022; Liang et al., 2022). It leads to metabolic disorders such as diabetes mellitus and obesity (Díez-Sainz et al., 2022; Cuevas-Sierra et al., 2019). Therefore, intestinal microbiota exocytosis and GDM may be related. However, there is very little evidence regarding the function of intestinal microbiota in the pathogenesis of HFD-induced GDM. Understanding the mechanism underlying the regulatory function of the intestinal microbiota and its metabolites in extracellular vesicles is essential for the precise control of GDM and obtaining valid clinical data to inform future therapeutic strategies.

Studies have found inconsistent results regarding the intestinal microbiota of women with GDM (Sun et al., 2023; Vavreckova et al., 2022; Yan et al., 2022; Liu et al., 2023), and few scholars have conducted joint analyses of their microbiota and metabolites. Therefore, we aim to establish an HFD-induced GDM mouse model and compare it with pregnant mice with normal blood glucose levels in this study; the results of multiregional sequencing of the bacterial community 16S rRNA and metabolites in their intestinal fecal exosomes were analyzed to provide targets for the diagnosis of GDM and therapeutic strategies.

## 2 Methods

### 2.1 Animal modeling

**Figure 1:**
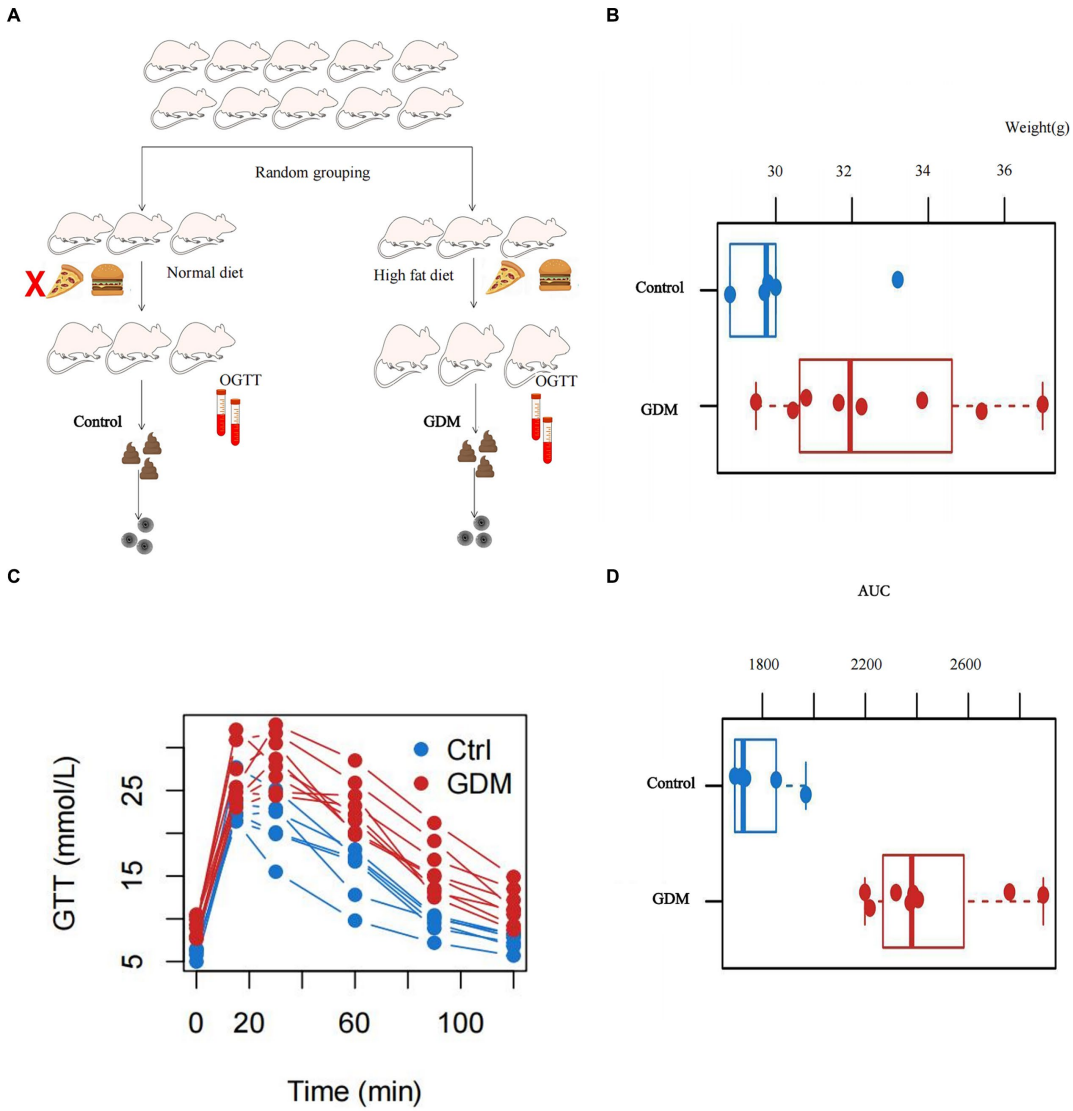
Basic characteristics of the pregnant mice. **(A)** Flowchart of the inclusion of mice where we constructed control and GDM groups of pregnant mice by feeding them different diets. **(B)** The body weights of the two groups of mice measured at the time of successful modeling, and the difference was statistically significant at *p* < 0.05. **(C)** The trend of the blood glucose profile of the glucose tolerance test (GTT) in the two groups of mice. **(D)** The area under the curve (AUC) plotted against the GTT results of the two groups of mice, and *p* < 0.05 was statistically significant.

The study was approved prior to implementation by the Ethics Committee of the Second Affiliated Hospital of Fujian Medical University, where the investigators were located (approval number: 2023; Ethical review no. 289). Healthy 6-week-old female C57BL mice (weighing 14.37 ± 0.66 g) were housed in a room at 22–26°C, with a darkness/light period of 12 h/12 h and constant air circulation. The mice were acclimatized with normal feed and water for 1 week. The mice were mated with C57BL male mice of the same age that had been fed a normal diet. The morning thereafter, the female mice were examined for vaginal mucus plugs that represent day 0 of gestation (Liang et al., 2010). Then randomly divided into two groups. The two groups were fed an HFD and a normal diet, respectively. The GDM group was fed an HFD and water *ad libitum*, whereas the control group was fed a normal diet and water *ad libitum* (see Supplementary Table S1, composition of the feed fed to the two groups of mice). Blood glucose levels and body weight were measured and recorded on days 0, 5, 10, 15, and 19 of gestation. GTT was performed on day 15 of gestation, and mice in the GDM group with abnormal glucose tolerance were identified as successful models. Eight mice from the GDM group and six from the control group were included in this study. Feces were collected from all pregnant mice in both groups and placed in sterile containers, stored on dry ice, shipped to the central laboratory, and stored at −80°C prior to experimental processing (see Figure 1A). All the experimental protocols were performed according to relevant guidelines and regulations.

### 2.2 Experimental procedure

Exosomes were extracted from feces by ultracentrifugation, observed under a transmission electron microscope, and subjected to particle size fluorescence labeling and nanoflow detection (ultracentrifuge, Hitachi, CP100MX; transmission electron microscopy, Hitachi, HT-7700; particle size analyzer, NanoFCM, N30E). Subsequently, 16S rRNA sequencing and metabolite co-analyses were completed. 16S polycistronic sequencing was performed using the Illumina NovaSeq 6000 sequencing platform for super-resolved phage (S-MURF/5R) identification. A fragment of the bacterial ribosomal small subunit genome was identified and used to determine bacterial species and abundance [the Greengenes (GG) 16S rRNA gene database: May 2013 version, the cutoff parameters for identifying or grouping OTUs/ASVs: 99%]. High-resolution untargeted metabolomic analyses were performed using an AB Triple TOF 6600 mass spectrometer (AB SCIEX). The metabolites in the samples were structurally identified by matching information from local databases regarding the metabolite retention times, secondary fragmentation spectra, molecular masses (molecular mass error < 10 ppm) and collision energies. The identification results were subjected to strict manual secondary checks and confirmations.

### 2.3 Statistical analysis

The distribution of phyla in each sample was evaluated using α diversity analysis. The β diversity was analyzed by principal coordinates analysis based on “Jaccard distance” in R (version 2.5-6). Bacterial groups with significant differences in abundance between groups at different levels, such as phylum, order, genus, and class, were analyzed using the Wilcoxon Rank Sum Test. Metabolic function and functional enrichment analyses of gut microorganisms were performed using FAPROTAX software (Louca et al., 2016). Metabolite data analysis mainly included differential metabolite screening and correlation statistics. Then, the correlation between the metabolites and microbiota was analyzed. *p* < 0.05 can be judged as statistically significant.

### 2.4 Role of the funding source

The research sponsoring organization only sponsored the funds that needed to be spent on this research process and was not involved in the research design, collection and analysis of data, writing of the article, etc.

## 3 Results

### 3.1 Basic characteristics of the pregnant mice

Figure 1A shows the flow chart for pregnant mice. The body weight of the GDM group in this study was 31.98 ± 2.05 g, and that of the control group was 29.52 ± 2.54 g. The difference in body weight between pregnant mice in the GDM and control groups was statistically significant by the Wilcoxon rank sum exact test (*W* = 8, *p* < 0.05; see Figure 1B). A GTT was performed in all pregnant mice. The mice in the GDM group had significantly higher blood glucose levels than the control group not only in fasting blood glucose but also at different time points after glucose injection (see Figure 1C). The area under the curve (AUC) was plotted by Wilcoxon’s rank sum exact test, and the difference was statistically significant in the AUC between the GDM and control groups was found (*W* = 0, *p* < 0.05; see Figure 1D).

### 3.2 Differences in intestinal microbiota

#### 3.2.1 α diversity

**Figure 2:**
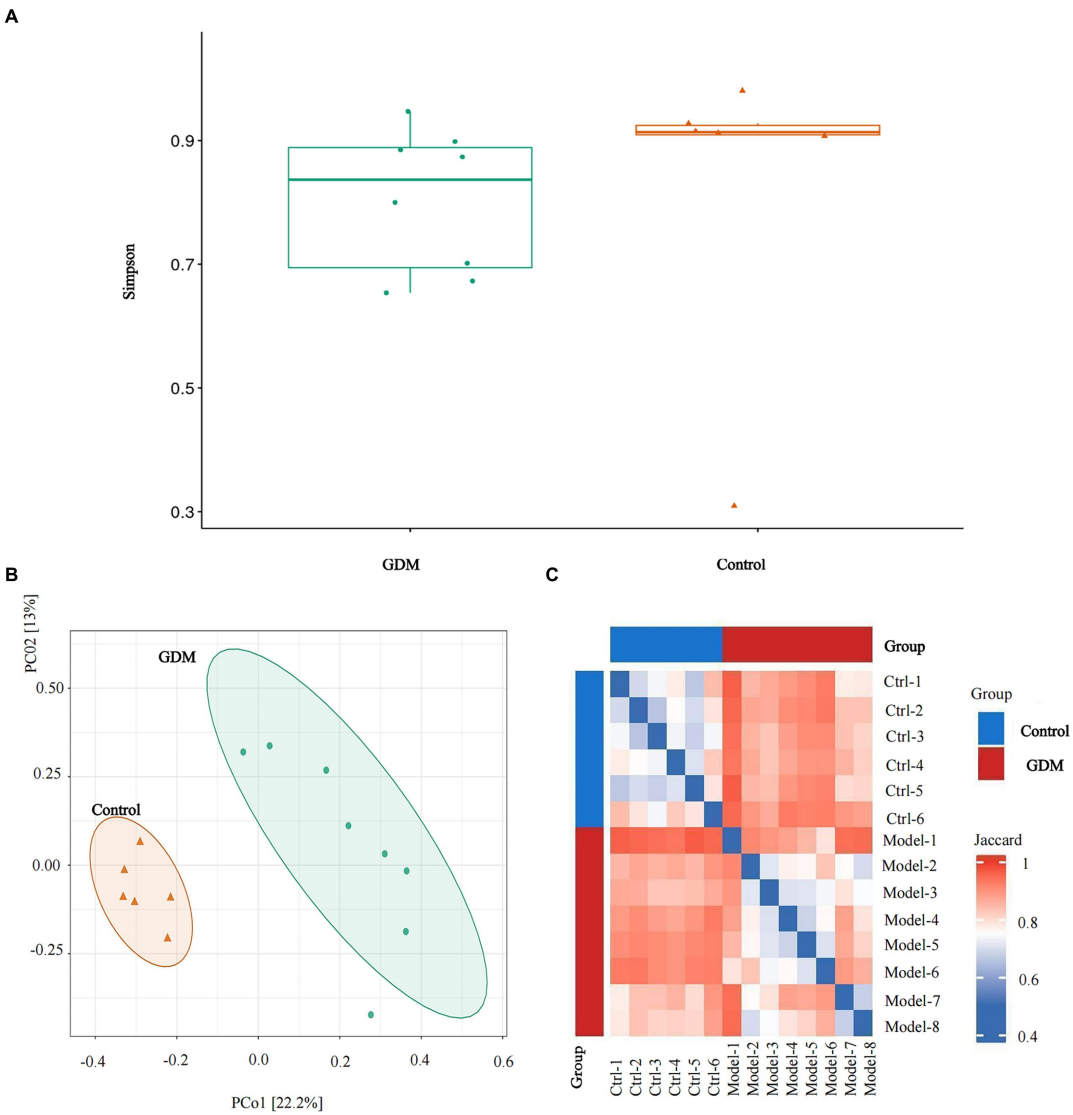
α diversity and β diversity of the two groups. **(A)** Simpson analysis of the α diversity of the two groups of intestinal microbiota. **(B)** Analysis of principal coordinates based on “Jaccard distance” through the R language. **(C)** The comparative analysis of the β diversity of the intestinal microbiota of the two groups. Relatively, red indicates a greater percentage of rise, and blue indicates a smaller percentage of rise.

The α diversity analysis using Simpson in the two groups, conducted with Wilcoxon’s rank sum exact test, revealed that diversity within the GDM group was reduced (*p* > 0.05; see Figure 2A).

#### 3.2.2 β diversity

Analysis of principal coordinates based on “Jaccard distance” in R showed that specimens in the GDM group were dispersed and varied more than those in the control group (see Figures 2B,C).

#### 3.2.3 Differences in the abundance of intestinal microbiota

**Figure 3:**
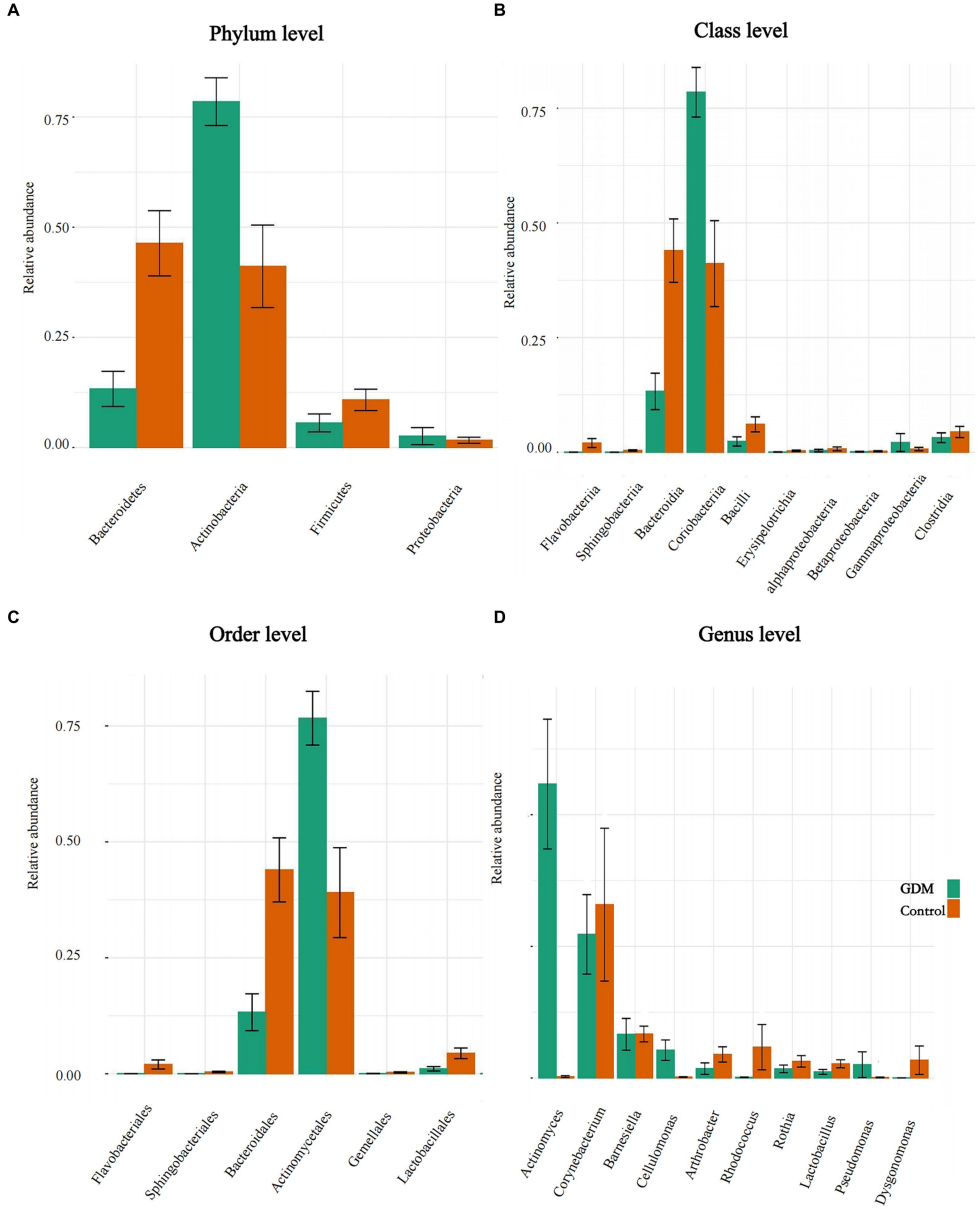
Differences in abundance of intestinal microbiota. **(A–D)** The level differences in relative abundance of intestinal microbiota between the GDM and control groups. **(A–D)** The difference in the microbiota at phylum, class, order, and genus level, respectively.

Actinobacteria was significantly more abundant in the GDM group than in the control group at the phylum level, accounting for approximately 75% of the total abundance of all microbiota in the GDM group, which was nearly twice that in the control group. However, the relative abundances of Bacteroidetes and Firmicutes were significantly lower than in the control group. Bacteroidetes had the largest difference in bacterial abundance in the two groups. The relative abundance of Bacteroidetes in the GDM group was one-third of the control group. Additionally, the relative abundance of Proteobacteria was higher than that of the control group. All these results were statistically significant (*p* < 0.05; see Figure 3A).

In the GDM group, the relative abundance of Coriobacteriia increased at the class level, whereas Bacteroidia decreased. Additionally, various bacteria, such as Flavobacteriia, Bataproteobacteria, and Bacilli, were reduced in the GDM group. All results were statistically significant (*p* < 0.05; Figure 3B).

The abundance of Lactobacillales was lower in the GDM group than in the control group at the order level. The two orders with large differences in relative abundance were Actinomycetales and Bacteroidales. Actinomycetales had the highest abundance among the bacterial groups in the GDM group, which was twice as high as that in the control group. However, Bacteroidales was the most abundant order in the control group; it was nearly three times more abundant than that in the GDM group. Flavobacteriales were slightly less abundant in the GDM group. All the above results were statistically significant (*p* < 0.05; see Figure 3C). However, compared in the control group, the abundance of Bifidobacteriales was higher (*p* > 0.05).

*Lactobacillus* and *Rothia* were also significantly lower in the GDM group at the genus level. *Actinomyces* had the highest abundance in the GDM group, which was higher than that in the control group and was the most pronounced among bacterial groups. *Corynebacterium* and *Rhodococcus* showed opposite trends in the GDM group, with higher abundance in the control group. However, the abundance of *Cellulomonas* was significantly higher in the GDM group. These results were statistically significant (*p* < 0.05; Figure 3D).

#### 3.2.4 Differences in metabolic functions of intestinal microbiota

**Figure 4:**
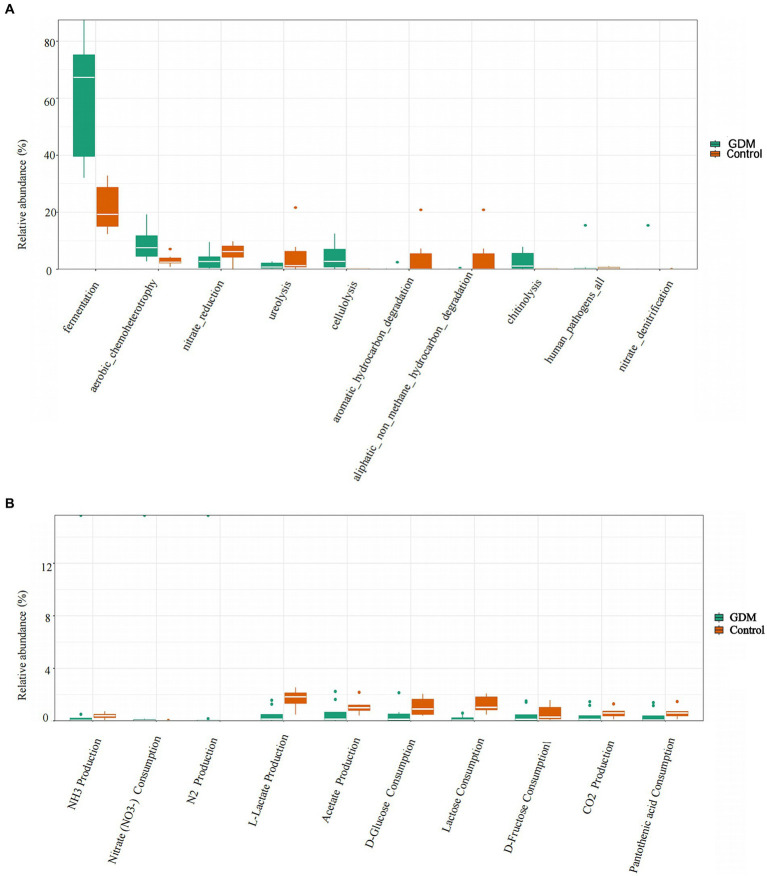
Differences in metabolic functions of intestinal microbiota. **(A)** The comparison of differences in function in two groups of intestinal microbiota. The control group is indicated in orange, and the GDM group in green. **(B)** The comparison of differences in metabolic function in two groups of intestinal microbiota. The control group is indicated in orange, and the GDM group in green.

We used FAPROTAX software to predict metabolic function in both groups. Differences in metabolic functions between the GDM and control groups were analyzed by the Wilcoxon Rank Sum Test, which revealed that fermentation, aerobic chemoheterotrophy, and cellulolysis in the GDM group were higher (*p* < 0.05). In contrast, nitrate reduction and ureolytic function decreased; however, the results were not statistically significant (*p* > 0.05). These results indicate that the genera related to nutrient metabolism underwent significant changes (see Figure 4A).

In this study, we found that nitrate consumption in the GDM group was significantly higher (*p* < 0.05). However, lactose consumption and L-lactate production were higher in the control group (*p* < 0.05) after between-group analysis using the Wilcoxon Rank Sum Test. Additionally, acetate production and D-glucose consumption were lower in the GDM group, although they were not statistically significant (*p* > 0.05; see Figure 4B).

### 3.3 Expression of metabolites of intestinal microbiota in exosomes

#### 3.3.1 Overall differential expression of metabolites

**Figure 5:**
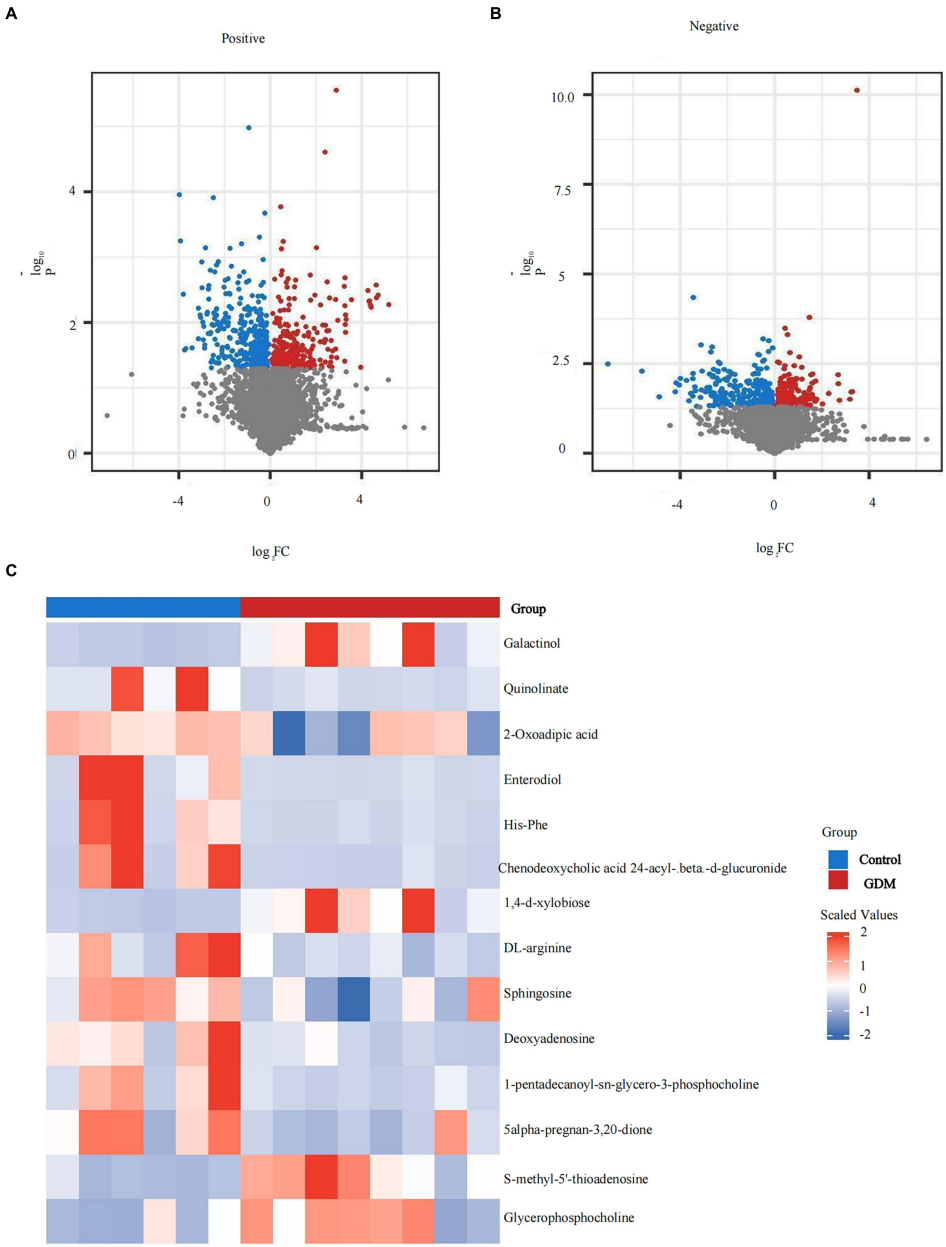
Expression of metabolites of intestinal microbiota in exosomes. **(A)** The positive ion metabolite volcano plots for both groups of colonies. **(B)** The negative ion metabolite volcano plots for both groups of colonies. Differences in metabolite expression (log2 fold change) were used as the horizontal coordinate, and the statistical significance (*p*-value of −log10) was used as the vertical coordinate. **(C)** The specific distribution of metabolites in the two groups. Metabolites in red are elevated, and metabolites in blue are decreased.

Significant differentially expressed metabolites were screened and selected, and volcano plots were visualized using the “ggplot2” package in R. Horizontal coordinates are gene expression differences (log2 fold change), and vertical coordinates are statistically significant (*p*-value of −log10). Fold Change > 1.5, *p* value < 0.05 metabolites are shown in red, and fold change < 0.67, *p* value < 0.05 metabolites are shown in blue. Non-significantly different metabolites are indicated in gray. The distributions of positive and negative metabolites in the GDM and control groups are shown (*p* < 0.05; see Figures 5A,B).

#### 3.3.2 Specific distribution of metabolites

Overall, the levels of positive ion mode metabolites, including sphingosine, DL-arginine, deoxyadenosine, 1-pentadecanoyl-sn-glycero-3-phosphocholine, and 5-alpha-pregnan-3,20-dione, were higher in the control group, whereas S-methyl-5′-thioadenosine and glycerophosphocholine were higher in the GDM group (*p* < 0.05). Negative ion pattern metabolites enterodiol, His-Phe, quinolinate, 2-oxoadipic acid, and chenodeoxycholic acid 24-acyl-betaD-glucuronide levels were lower (*p* < 0.05) in the GDM group. However, galactinol and 1,4-d-xylobiose levels were higher (*p* < 0.05). Overall, compared with the control group, GDM mice showed a decrease in the expression of most metabolites in exosomes and an increase in the expression of a few metabolites (see Figure 5C).

### 3.4 Correlation between intestinal microbiota and metabolites

**Figure 6:**
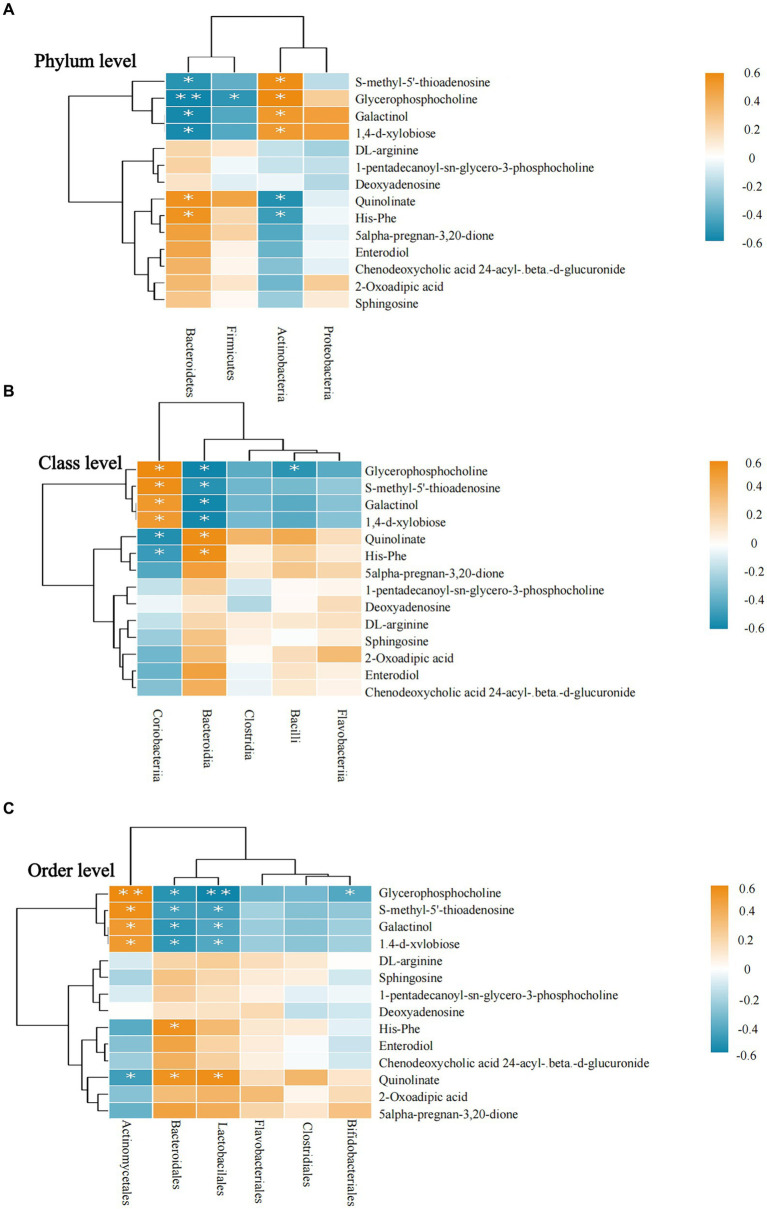
Correlation between intestinal microbiota and metabolites. **(A–C)** Intestinal microbiota and metabolite correlations. **(A)** The flora-metabolite correlation at the phylum level. **(B)** The correlation of microbiota with metabolites at the class level. **(C)** The correlation of microbiota with metabolites at the order level. Orange color indicates a positive correlation between intestinal microbiota and metabolite levels, whereas green color indicates a negative correlation, and * indicates a statistically significant difference in the correlation between microbiota and metabolites.

Correlation analyses revealed that Actinobacteria was positively correlated with S-methyl-5′-thioadenosine, glycerophosphocholine, galactinol, and 1,4-d-xylobiose at the phylum level and negatively correlated with quinolinate and His-Phe. In the correlation analyses of these metabolites, Bacteroidetes showed opposite trends to those of Actinobacteria. All results were statistically significant (*p* < 0.05; see Figure 6A).

At the class level, Bacteroidia was negatively correlated with glycerophosphocholine, S-methyl-5′-thioadenosine, galactinol, and 1,4-d-xylobiose and positively correlated with quinolinate and His-Phe. Among these six metabolites, the correlation results were completely opposite for Coriobacteriia and Bacteroidia. All results were statistically significant (*p* < 0.05; see Figure 6B).

At the order level, Bifidobacteriales was negatively correlated only with glycerophosphocholine, whereas Bacteroidales and Lactobacillales showed a more consistent trend regarding their correlation with metabolites and had positive correlations with glycerophosphocholine, S-methyl-5′-thioadenosine, galactinol, 1,4-d-xylobiose. However, Actinomycetales showed the opposite correlation. Similarly, quinolinate was positively correlated with Bacteroidales and Lactobacillales and negatively correlated with Actinomycetales. All results were statistically significant (*p* < 0.05; see Figure 6C).

**Figure 7:**
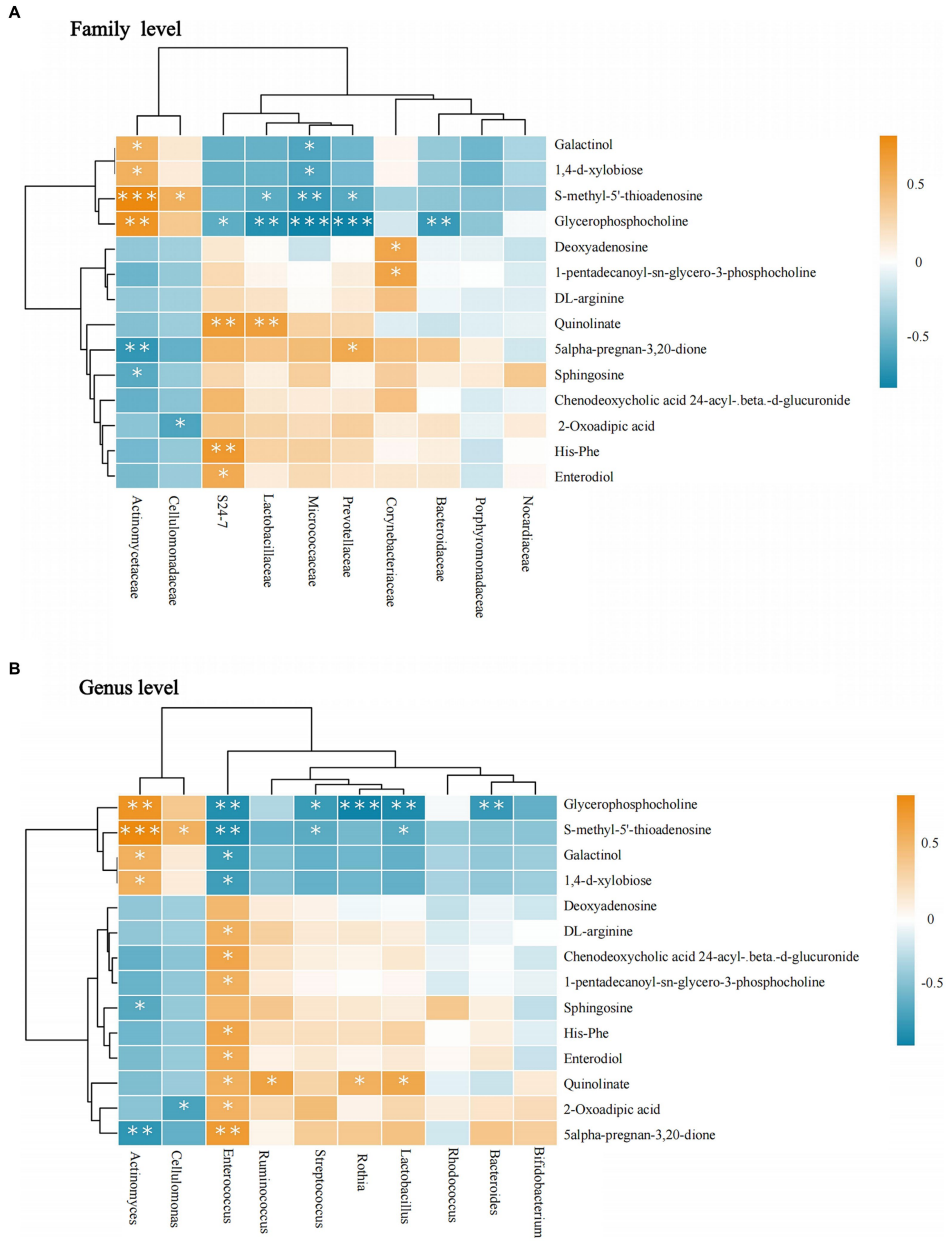
Correlation between intestinal microbiota and metabolites. **(A)** The flora-metabolite correlation at the family level. **(B)** The flora-metabolite correlation at the genus level. Orange color indicates a positive correlation between intestinal microbiota and metabolite levels, whereas green color indicates a negative correlation, and * indicates a statistically significant difference in the correlation between microbiota and metabolite.

At the family level, Actinomycetaceae was positively correlated with most metabolites, glycerophosphocholine, S-methyl-5′-thioadenosine, galactinol, 1,4-d-xylobiose, whereas they were negatively correlated with sphingosine and 5-alpha- pregnane-3,20-dione. Lactobacillaceae was negatively correlated with glycerophosphocholine and S-methyl-5′-thioadenosine. In contrast to Actinomycetaceae, Micrococcaceae were negatively correlated with glycerophosphocholine, S-methyl-5′-thioadenosine, galactinol, and 1,4-d-xylobiose. Prevotellaceae was negatively correlated with glycerophosphocholine and S-methyl-5′-thioadenosine and positively correlated with 5-alpha-pregnan-3,20-dione. Corynebacteriaceae had a positive correlation with deoxyadenosine and 1-pentadecanoyl-sn-glycero-3-phosphocholine. Bacteroidaceae had a negative correlation with glycerophosphocholine (see Figure 7A).

At the genus level, *Actinomyces* and *Cellulomonas* showed consistent trends. *Actinomyces* showed a positive correlation with glycerophosphocholine, S-methyl-5′-thioadenosine, galactinol, and 1,4-d-xylobiose and negative correlations were observed for sphingosine and 5-alpha-pregnan-3,20-dione. *Enterococcus* was positively correlated with the highest number of metabolites, in contrast to *Actinomyces*. Additionally, quinolinate was positively correlated with *Ruminococcus*, *Rothia*, and *Lactobacillus*. S-methyl-5′-thioadenosine was negatively correlated with *Lactobacillus* and *Streptococcus*. Glycerophosphocholine had a negative correlation with *Bacteroides*, *Lactobacillus*, *Rothia*, and *Streptococcus* spp. All results were statistically significant (*p <* 0.05; Figure 7B).

## 4 Discussion

**Figure 8:**
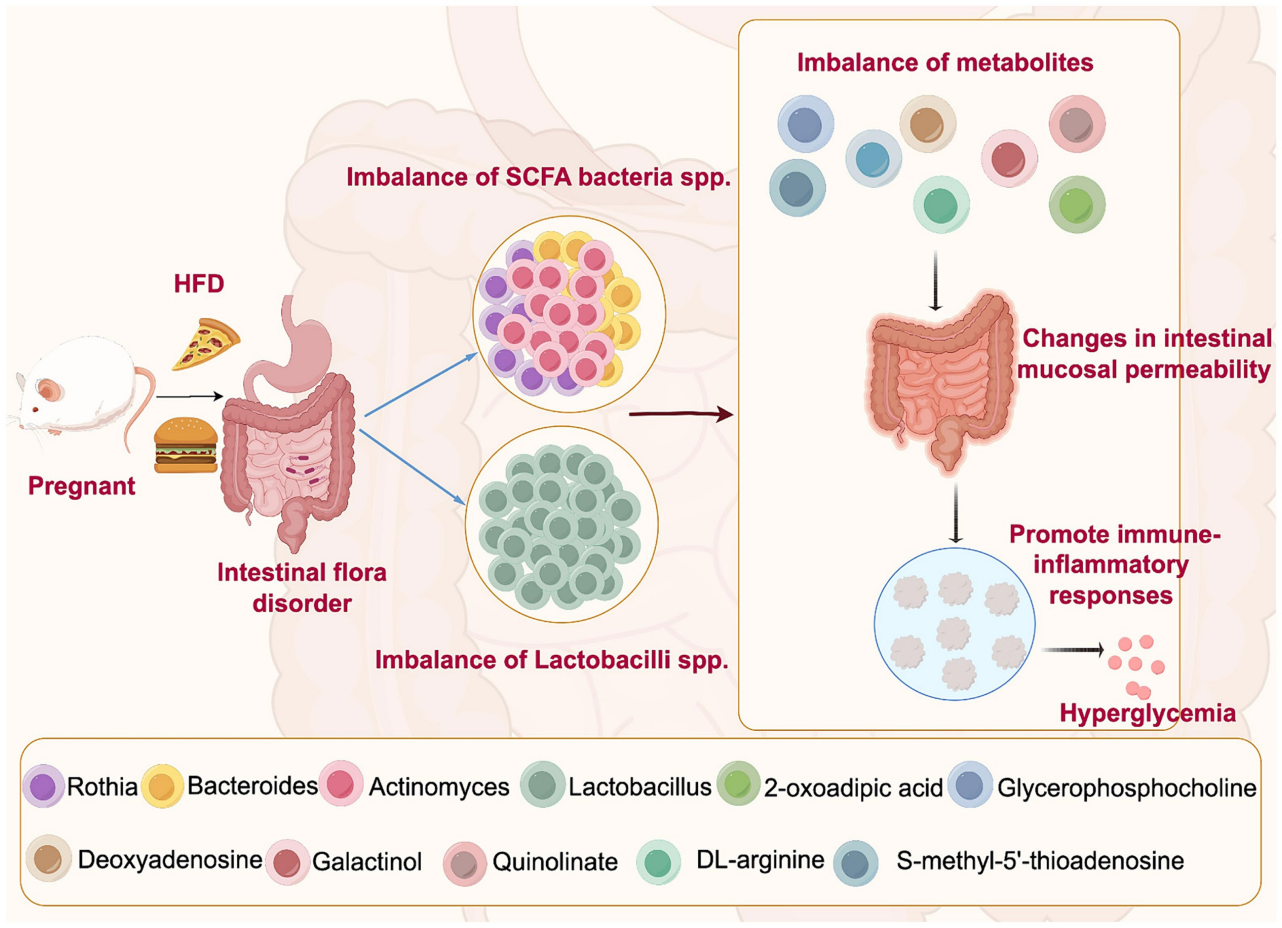
Mechanisms of high-fat diet-induced intestinal microbiota disruption leading to GDM. High-fat diet may lead to the disorder of intestinal microbiota of SCFA bacteria spp. and *Lactobacillus* spp., resulting in the imbalance of their metabolites. Due to their impact on intestinal barrier function, they may cause changes in intestinal mucosal permeability and promote immune-inflammatory responses, which can aggravate the fluctuations in blood glucose levels, leading to the onset and development of GDM.

Our results show the differences and correlations between bacterial flora and their metabolites in the intestinal fecal exosomes of an HFD-induced GDM mouse model and healthy pregnant mice for the first time. A range of intestinal microbiota and metabolites associated with GDM were identified, particularly, the SCFA-fermenting bacterial species, *Lactobacillus* and *Actinomyces* (see Figure 8). For GDM, altering dietary intake to modulate the intestinal microbiota and metabolites may be a promising therapeutic strategy.

### 4.1 HFD alters intestinal microbiota in pregnant mice

Studies have found the abundance of SCFA-producing genera is reduced in patients with GDM (Ye et al., 2023). *Bacteroides*, *Bifidobacterium*, *Prevotella,* and *Streptococcus*, among others, are SCFA-producing bacteria (Ye et al., 2023; Serban et al., 2023). However, previous studies did not specify the diet for GDM; therefore, current evidence on the role of an HFD in GDM remains limited. Our study found the role of SCFA-producing bacteria in HFD. Owing to their impact on intestinal barrier function, they may lead to alterations in intestinal mucosal permeability and promote immune-inflammatory responses, which may aggravate fluctuations in blood glucose levels, leading to the onset and progression of GDM (Zhang et al., 2022). Actinobacteria had the highest abundance in the GDM group at the phylum level. It was much higher than that in the control group. The researchers found that the relative abundance of Bacteroidetes was lower in both GDM as well as obese patients (Ding et al., 2021; Grigorescu and Dumitrascu, 2016), which is in line with our findings regarding the HFD GDM model group.

*Lactobacillus* was significantly lower in the GDM group at the genus level. A systematic evaluation showed that *Lactobacillus* was lower in women with GDM than in healthy pregnant women (Ding et al., 2021). However, women with GDM consuming an HFD have not been included in previous studies; thus, this research provides information on a correlation between HFD and GDM intestinal microbiota. The intake of probiotics, including *Lactobacillus*, for 12 weeks significantly reduced HbA1c, high-sensitivity c-reactive protein, fasting blood glucose, and total cholesterol levels in patients with GDM (Hasain et al., 2022). The lower abundance of *Lactobacillus* in the GDM model group may be due to the fact that less *Lactobacillus* is not conducive to glycemic control, leading to the development of insulin resistance in GDM. In future, we will search for the best probiotic strains and dosages for the treatment of GDM, which will help in the development of a therapeutic tool for GDM.

Additionally, we found that in the GDM group, *Rothia* was lower, whereas *Actinomyces* showed the opposite trend. Evidence from studies related to the intestinal microbiota in patients or animal exosomes receiving an HFD remains lacking. In patients diagnosed with GDM at 24–28 weeks of gestation, the researchers found a decrease in the intestinal microbiota of *Rothia* and *Actinomyces* in early pregnancy compared to healthy pregnant women (Hu et al., 2021). However, *Rothia* was higher in GDM women in late pregnancy (Crusell et al., 2018), making it difficult to determine its role in the pathogenesis of GDM. Future studies could measure the dynamics of *Rothia* over the gestational cycle. In contrast, *Actinomyces* seems to be a causative factor of GDM, which is reflected not only in the intestinal microbiota (Liu et al., 2019) but also in the oral microbiota (Yao et al., 2019). GDM increases the number and detection rate of oral microbiota. Compared in healthy pregnant women, *Actinomycetes* was significantly higher (Yao et al., 2019). We further confirmed this in our HFD-induced GDM model. Currently, the correlation results are inconsistent across studies, and more studies with large samples are needed to confirm this in the future.

### 4.2 HFD alters metabolic function in pregnant mice

We performed a functional analysis of bacterial flora metabolites in intestinal fecal exosomes from pregnant mice and found that microbial fermentation was higher in the GDM group. Study showed blackberry fermentation with fiber increased the levels of Bacteroidetes and decreased the levels of Firmicutes. Our study suggests that HFD-induced GDM in mice lead to differential fermentation of the intestinal microbiota, which may be a key cause of the altered abundance of Bacteroidetes. Dietary fiber can promote Bacteroidetes fermentation and glucose metabolism. An HFD may lead to abnormal glucose metabolism and insulin resistance in pregnant mice, possibly through high levels of fermentation by *Actinomyces*.

The main metabolites produced by gut microorganisms fermenting indigestible carbohydrates are SCFAs containing acetate, butyrate, and propionate (Ye et al., 2023; Louis and Flint, 2017). Acetate is mainly derived from the fermentation of dietary fiber by intestinal microbiota. It reduces the incidence of type 1 diabetes in mice (Daïen et al., 2021). However, evidence on GDM remains scarce. We found that the level of acetate in the GDM group was lower than in the control group, although no statistical significance was found; however, this does not mean that its role can be ignored. Bacteroidetes may be associated with acetate production in GDM (Sun et al., 2023). The control group in this study consumed more dietary fiber than the HFD, which may promote the production of acetate through bacterial fermentation by SCFA-producing genera, such as Bacteroidetes, whereas the opposite is true for Actinobacteria. Lower acetate levels in GDM may lead to poorer glycemic control than in healthy mice.

Lactose consumption and L-lactate production were lower in the GDM group. SCFA produced by bacterial fermentation are the main source of energy in animals (Iwanaga and Kishimoto, 2015). Under conditions of reduced glucose utilization, ketone bodies and lactate play key functions as alternative energy substrates. It has been suggested that *Lactobacillus*, *Enterococcus*, and *Bifidobacterium* produce lactic acid by fermenting carbohydrates (Castro et al., 2016). An acidic environment is created in the intestines, which is not conducive to the growth of harmful bacteria. And *L. rhamnosus* LRa05 was found to improve insulin resistance in type 2 diabetic mice (Wu et al., 2021). In this research, the decrease in *Lactobacillus* in the GDM group, with a decrease in the level of fermented carbohydrates and a parallel decrease in the lactic acid produced, may have created an environment for the growth of harmful bacteria, leading to dysbiosis and abnormal glucose metabolism. However, evidence related to intestinal microbiota remains scarce. More studies are needed to confirm these results in HFD-induced GDM.

Additionally, nitrate consumption was significantly higher in the GDM group than in the control group (*p* < 0.05). In contrast, nitrate reduction in the GDM group was lower; however, this result was not statistically significant (*p* > 0.05). Studies have shown that nitrates increase the risk of GDM in the second trimester of pregnancy (Yu et al., 2020). However, research on nitrate levels in GDM remains limited. Most studies have focused on the relationship between nitrate and oral microbiota rather than intestinal microbiota. *Rothia* reduces nitrate levels in the oral cavity (Liddle et al., 2019). And nitrate reduction leads to dysbiosis of the oral microbiota, which affects metabolism and thus increases the risk of diabetes mellitus (Morou-Bermúdez et al., 2022).

The interaction of intestinal microbiota with nitrate may be similar to that of oral microbiota in mice with GDM in our study. The difference in *Rothia* abundance may have contributed to the higher nitrate intake in the GDM group. *In vitro*, nitrate stimulates lactate production by nitrate-reducing bacteria, wherein lactate is initially oxidized to pyruvate. It is then oxidized to weaker acids such as propionate and acetate (Wicaksono et al., 2020). In addition, dietary nitrate supplementation can lead to a decrease in *Actinomyces* in the oral cavity (Burleigh et al., 2019). In this research, HFD may lead to the disruption of oral microbiota, such as *Rothia* and *Actinomyces*, affecting the intestinal microbiota through the digestive tract. This may partly explain the occurrence of GDM.

### 4.3 Relationship between metabolites and intestinal microbiota in pregnant mice

There is a correlation between purine metabolic pathways and diseases such as coronary heart disease, and diabetes (Lu et al., 2016). The purine metabolites include deoxyadenosine, adenosine, guanosine-5′-monophosphate, and adenosine-5′-monophosphate. *Streptococcus* can synthesize deoxyadenosine to suppress the host immune response (Dai et al., 2018). Additionally, 3-deoxyadenosine altered the abundance of intestinal bacteria such as Firmicutes and Bacteroidetes in obese rats induced by a HFD and reduced kidney and epididymal fat and body weight (An et al., 2018). We found a positive correlation between Corynebacteriaceae and deoxyadenosine. Corynebacteriaceae may be involved in the pathogenesis of GDM through deoxyadenosine synthesis. Additionally, *Actinomyces* and *Cellulomonas* may contribute to GDM through the production of S-methyl-5′-thioadenosine, whereas *Enterococcus*, *Streptococcus*, and *Lactobacillus* may reduce S-methyl-5′-thioadenosine. These findings provide strong evidence of an interaction between purine metabolism and bacterial flora in GDM.

Evidence between DL-Arginine and intestinal microbiota in GDM is still lacking. Hu et al. (2022) have demonstrated in their research that the occurrence of chronic hyperglycemia may be because DL-arginine inhibits the inflammatory response and reduces the levels of blood glucose and advanced glycosylation end products (AGEs) in rats with STZ-induced T2 DM. It plays a key role in the mid-late phase by downregulating AGEs receptor-1 and upregulating glucose transporter protein 4 expression in the liver. We found that *Enterococcus* in the GDM group was positively correlated with DL-arginine, which may promote DL-arginine production as a valuable biomarker for GDM.

Wu’s study (Wu et al., 2022) found a significant downregulation of galactinol, which may be related to D-arginine metabolism in T2 DM and diabetic nephropathy animal models. In our study, galactinol was associated with several different levels of microbiota which may be a key metabolite in GDM. *Actinomycetes* may promote their production, whereas *Enterococcus* may hinder it. Although elevated galactinol levels may be related to GDM, unlike the findings in T2 DM, there are few relevant studies on GDM, and further validation is required.

Quinolinate was positively associated with the risk of developing type 2 diabetes and was associated with Firmicutes and Bifidobacterium (Qi et al., 2022). Quinolinate, kynurenate, kynurenine, and xanthurenate can be metabolized via the kynurenine pathway (Qi et al., 2022). This pathway plays a key role in insulin resistance, immune activation, and inflammation (Badawy, 2017), but its mechanism in the intestinal microbiota is unclear. In our study, quinolinate was positively associated with Bacteroidetes*, Lactobacillus*, *Rothia*, *Ruminococcus*, and *Enterococcus* and negatively correlated with Actinobacteria. The consumption of fiber-rich foods is the dietary factor most closely related to tryptophan metabolites rather than protein/tryptophan-rich foods (Qi et al., 2022). The GDM group in our study consumed an HFD, whereas the control group had a relatively high fiber content in their diet, and the GDM group had a low quinolinate content, suggesting that the effect of quinolinate on GDM may be different from that on T2 DM.

Our results show that the GDM group exhibited higher abundance in *Actinomyces* and lower levels of sphingosine. Sphingosine was negatively correlated with *Actinomyces*. It has been shown that an HFD affects host lipids by altering *Streptococcus* and *Eubacterium coprostanoligenes*. The combination of sphingosine with these two genera modulates lipid levels in HFD mice (Wei et al., 2021). For GDM, a chronic HFD may lead to the development of obesity and hyperlipidemia, which may be caused by the inability of *Actinomyces* to regulate lipid levels in pregnant mice due to the inhibition of sphingosine production by *Actinomyces*.

The abundance of 2-oxoadipic acid in the GDM group was lower than in the control group. It was positively correlated with *Enterococcus* and negatively correlated with *Cellulomonas*. However, research on the relationship between 2-oxoadipic acid and *Enterococcus* spp. is lacking. It was found that metabolites, such as 2-oxoadipic acid and carbohydrate metabolites may be higher in the pig cecum after long-term intake of high-fiber diets. Bacteroidetes increased with the increased intake of a high-fiber diet (Wu et al., 2021). High-fiber diets alter the diversity and structure of bacterial flora and their metabolites. Based on our findings, *Enterococcus* may promote the production of 2-oxoadipic acid, whereas the opposite is true for *Cellulomonas*. The dietary fiber composition was higher in the control group, which explains the higher 2-oxoadipic acid levels. Although previous experiments have found higher levels of *Enterococcus* in patients with GDM (Ding et al., 2021), few relevant studies exist, and further research is required to verify this.

## 5 Limitations and future research directions

Our study, despite having a small sample size in animal models, clearly reflects the differences in intestinal microbiota and metabolites between the two groups. This adds new evidence on the intestinal exosome microbiota and metabolites in HFD-induced GDM in mice, which will be critical to discovering therapeutic and early diagnostic approaches to GDM in the future. More samples should be included for further in-depth studies in the future. In addition, pregnant women with GDM who consume HFD could be included and compared with the intestinal microbiota of healthy pregnant women. Due to research funding constraints, we did not measure the initial microbiota of both groups of mice prior to conducting their dietary intervention. Future studies could add such baseline measurements to help understand the initial microbiota composition as well as the changes that occur after dietary interventions and make study designs more robust.

## 6 Conclusion

In this study, we identified potential interactions of dietary factors on gut bacteria and their interactions with related metabolites. An imbalance in the production of SCFA, *Lactobacillus* spp., and the associated metabolites may contribute to GDM. GDM could be prevented and treated by modifying the diet and influencing changes in the intestinal microbiota in the future. Pregnant women can consult with a dietitian or physician to develop a personalized dietary plan that reduces the intake of saturated fats and increases the intake of unsaturated fats. Additionally, they can supplement with probiotics such as Lactobacillus to produce SCFAs, thereby helping to improve gut microbiota health and reduce the risk of GDM.

## Data Availability

The raw data supporting the conclusions of this article will be made available by the authors, without undue reservation.
