# Supplementary figures and images for: Suppression of certain intestinal microbiota metabolites may lead to gestational diabetes in mice fed a high-fat diet
Source: Front Microbiol. 2024 Sep 16;15:1473441. doi: 10.3389/fmicb.2024.1473441 (PMC11439706; doi:10.3389/fmicb.2024.1473441)

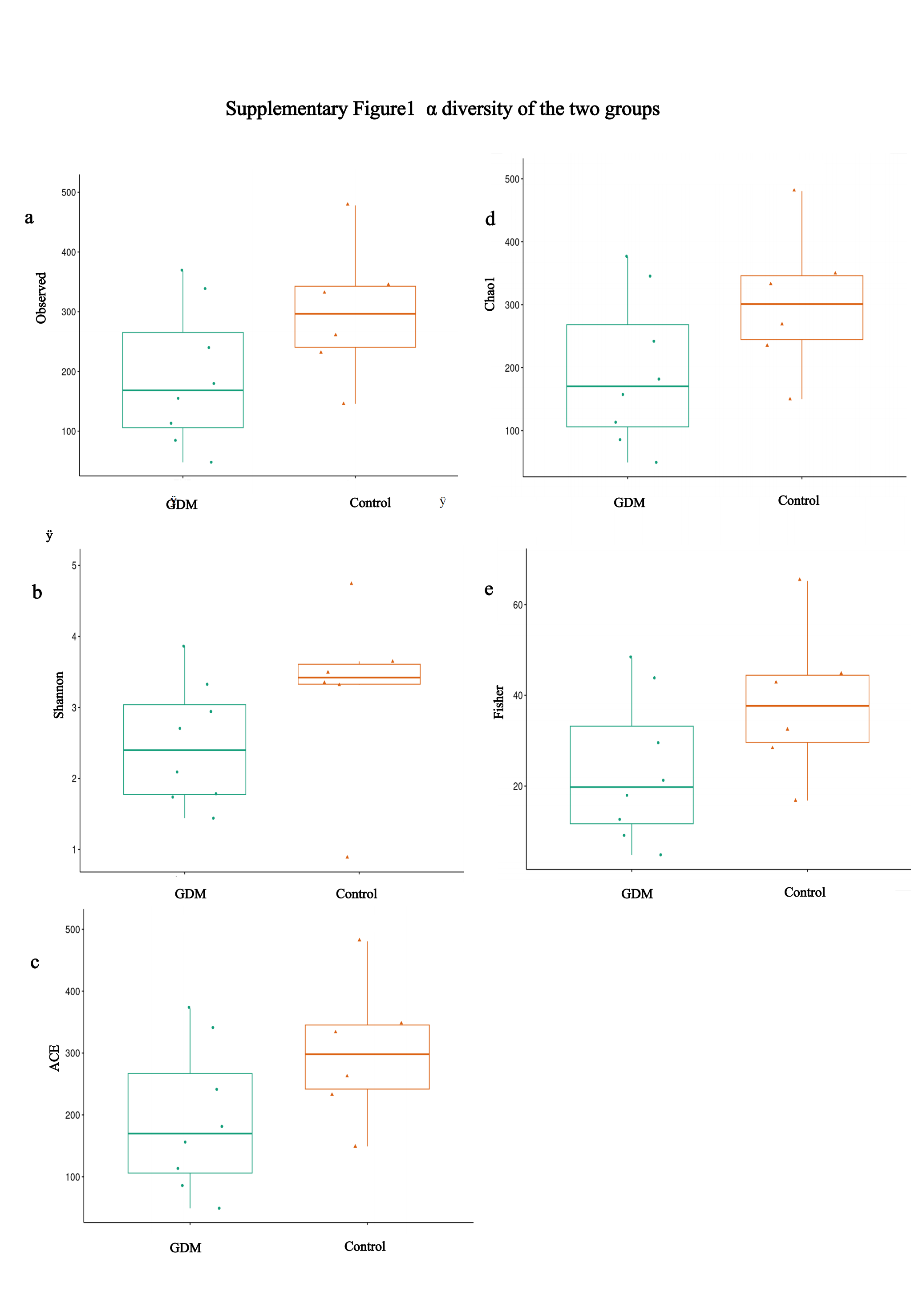

Supplement: Supplementary file 1 [file Image_1.TIF]
