# Supplementary material for: Suppression of certain intestinal microbiota metabolites may lead to gestational diabetes in mice fed a high-fat diet
Source: Front Microbiol. 2024 Sep 16;15:1473441. doi: 10.3389/fmicb.2024.1473441 (PMC11439706; doi:10.3389/fmicb.2024.1473441)
Supplement: Supplementary file 2 [file Table_1.DOCX]

**Supplementary Table 1 Composition of the feed fed to the two groups of mice**

| Group | High-fat Diet Feed (Model Group) | | Normal Diet Feed (Control Group) |
| --- | --- | --- | --- |
| Item | Proportion of mass | Proportion of energy | Proportion of energy |
| Fat | 35%(including 9.26% soybean oil and 90.74% lard. 46.84% saturated fats and 53.16% unsaturated fats) | 60% | 12.11%(all soybean oil. 15.9% saturated fats and 84.1% unsaturated fats) |
| Protein | 26% (casein, cysteine) | 20% | 22.47% (soybean meal, fish meal, lysine) |
| Carbohydrate | 26% (maltodextrin, sucrose, cellulose) | 20% | 65.42% (corn, wheat bran, wheat by-products) |
